# Supplementary material for: Are carbon emissions trading and green financial instruments synergistic? -Comprehensive quantitative research based on content analysis
Source: PLoS One. 2024 Mar 7;19(3):e0298601. doi: 10.1371/journal.pone.0298601 (PMC10919601; doi:10.1371/journal.pone.0298601)
Supplement: S1 Data — (ZIP) [file pone.0298601.s001.zip › Data/地级市Z_F工作报告.zip!/╔┬╬≈/║║╓╨╩╨/└┤╫╘╣1⁄2╓┌║┼í╛┬φ┐╦╩2╛▌═°í┐_2015.docx]

各位代表：

现在，我代表市人民政府向大会作工作报告，请予审议，并请市政协委员和其他列席人员提出意见。

一、四届人大五次会议确定任务完成情况

过去一年，全市上下认真贯彻中、省一系列决策部署，积极应对错综复杂的宏观经济形势，牢牢把握稳中求进、改革创新、提质增效的核心要求，以实施“六大战略”、培育“五大产业”为引领，以推进“十大工程”和建设“五大园区（村）”为抓手，奋发有为、苦干实干，较好地完成了四届人大五次会议确定的各项目标任务。

——经济保持较快增长。完成生产总值991亿元，增长11.6%，增速全省第三。规模以上工业总产值突破千亿元，增加值达到312.47亿元，增长17.1%，增速全省第三；农业总产值325.51亿元，增长5.4%，增速全省第二；社会消费品零售总额281.42亿元，增长13.5%，增速全省第四。

——结构调整成效显著。三次产业比例为18.6∶46.2∶35.2，结构更趋合理。猪茶菜产值占农业总产值的比重达到55.2%，轻、重工业比重由上年的25∶75调整为27.7∶72.3，装备制造、有色冶金、食品工业、能源化工的产值占规模以上工业总产值的比重达到85.7%。非公经济增加值占生产总值的比重为51.5%。——质量效益稳步提升。财政总收入90.95亿元、增长8.6%；地方财政收入40.89亿元、同口径增长15.55%，非税收入占比26.9%。规模以上工业产销率93.5%，主营业务收入和利税总额分别增长9.7%、19.4%。——人民生活明显改善。城镇居民人均可支配收入24605元、增长11%；农民人均纯收入7933元、增长12.5%。全年民生类支出194.3亿元，占财政总支出的83%，公共服务能力进一步增强。

一年来，我们着力抓了以下几方面工作：

（一）贯彻产业兴市战略，力促经济逆势增长。农业上，围绕市场调结构、转方式，培育壮大特色主导产业，大力实施“农业倍增工程”，粮、油总产分别达101.75万吨、18.91万吨；生猪出栏416.39万头，茶叶、蔬菜、水果产量分别达3.3万吨、214万吨和43万吨，汉中仙毫在国际茶博会上获27枚金奖。新增省、市级农业园区64个，新发展专业合作社569个、市级示范家庭农场105个。工业上，面对市场需求低靡、环境约束加剧等外部因素的严峻考验，坚持平茬齐过、盘点过堂、一企一策、精准发力，力促工业克难攻坚、提质增效、快速增长，“工业翻番工程”目标如期实现，规模以上工业总产值达到 1003.83亿元、增长16.6%。80个工业重点项目完成投资56亿元。新培育规模以上企业53户。新增省级工业园区5个。新增陕西名牌产品17个，创建省级知名品牌示范区2个。三产上，强力实施“全域旅游工程”，以旅游业快速发展牵动服务业持续提升。13个重点旅游项目完成投资9.2亿元，张骞墓申遗成功，新创建4A级景区4个、3A级景区2个，全年旅游收入131.4亿元、增长23.9%。汉中茶城建成运营，亿丰国际商贸城、普汇中金世界港等项目开工建设。全年新增限额以上贸易单位79户。外贸进出口和引进外资分别达1.04亿美元、4005万美元。金融机构人民币存、贷款余额分别增长11.5%、12.8%，贷存比较去年上升0.45个百分点。

（二）坚守生态立市战略，加快建设美丽汉中。以环境治理“四个战役”为重点，扎实开展汉江流域污染防治“三年行动”，“生态环境工程”取得新成效。实施污染治理项目121个，新建和改造污水管网910公里，清理城乡垃圾2.24万吨；封存和改造燃煤锅炉38台，机动车“油改气”3500多辆，淘汰报废“黄标车”、老旧车16000多辆；中心城区生活污水、垃圾处理率分别达91.2%、100%，县城分别达89.1%、98.1%；农作物秸秆禁烧率和综合利用率分别达到97%、71.3%。新开工节能重点工程10个，完成减排项目42个。关闭落后生产线6条，淘汰落后产能106万吨。完成造林19.2万亩，道路及河堤绿化492公里，治理水土流失850平方公里。全年节能减排任务超额完成，汉江出境水质保持在二类以上，中心城区空气质量二级以上优良天数355天（API标准）。

（三）推进文化名市战略，坚持不懈改善民生。大力实施“文化振兴工程”，加大文化产品扶持力度，推出了一批精品力作。专题片《汉源》、歌曲《油菜花儿开》获省“五个一工程”奖，剧目《汉颂》、《韩信拜将》获省第七届艺术节一等奖，我市在陕南民歌大赛上获得8项大奖；广播电视“户户通”4.3万户，免费开放文化（图书）馆223个，市羌文化中心主体完工。大力实施“民生改善工程”、“扶贫攻坚工程”。深化教育领域综合改革，宁强、佛坪荣获“国家义务教育发展基本均衡县”称号，西乡、留坝被命名为“双高双普”县，全市高考再创佳绩；营养改善计划惠及学生25.7万人，享受助学补贴9.2万人。市中心医院和中医院顺利整合、运行健康，县级公立医院综合改革稳步推进，89个公共卫生服务示范镇全部建成，县、镇、村医疗机构药品“三统一”实现全覆盖。“单独两孩”政策稳步实施，人口自然增长率2.42‰。新增就业3.54万人，农村居民进城落户10.4万人。全年申请专利1288件、授权914项，取得科技成果52项。城乡低保、农村五保、医疗和临时救助等政策得到全面落实，城镇医保、城乡养老参保率和新农合参合率均达99%以上。全面落实了27.5万高龄老人、5.8万名残疾人、1269名孤儿政策补贴。2014“环中赛”汉中段赛事成功举办，省十五届运动会获得金牌69枚。保障性住房基本建成4.16万套，陕南移民搬迁开工建房2.2万户，城乡15.7万人乔迁新居，保障房建设、移民搬迁工作在全省考评中均获一等奖；汉江综合整治工程建成堤防主体40.8公里。

（四）实施项目拉动战略，不断扩大投资规模。大力推进“千亿投资工程”，紧盯不放争跑项目，多路出击招商引资，狠下硬茬保障环境，固定资产投资规模持续扩大。全社会固定资产投资845.04亿元，增长24.4%；212个重点项目完成投资383.83亿元，增长31%。招商引资到位资金417亿元，增长19%。汉中航空智慧新城规划已经省上批准，省政府出台了支持建设的政策措施；汉中城固机场正式通航，运营情况良好；西成客专汉中段全线开工、路基工程完成75%，宝汉高速汉中以南段路基工程贯通、以北段全线开工，洋县桑溪铁矿资源整合通过省政府确认，阳安铁路扩能改造、众泰汉龙汽车项目开工建设，汉巴渝快速铁路已列入国家交通规划。

（五）强化城镇带动战略，统筹推进城乡发展。围绕建设生态宜居城市，大力实施“城乡统筹工程”，新型城镇化建设取得新突破。按照“五大城市形象设计”要求，精心编制了《城乡统筹发展规划》等20余项规划。中心城区49个城建重点项目完成投资38亿元，西二环中段、梁州路等项目基本建成，沙沿路等项目进展顺利，前进路西段等项目开工建设，滨江新区、兴元新区开发和东关、南关片区改造有序推进。县城基础设施重点项目完成投资53亿元，省、市级重点镇建设完成投资39.2亿元。“国卫”创建通过国家技术评估。

500个“美丽乡村”建设全面启动，建成新型社区、示范社区66个，城乡面貌焕然一新。

（六）突出创新驱动战略，深化改革释放活力。以全面深化改革为统揽，以创新体制机制为抓手，不断激发发展活力。市级行政审批事项减少198项、精简率38%；政府规范性文件废止28件、修订55件。加快食品药品监管体制改革，154个镇（街道办）监管所建设达标。全面推行商事登记制度改革，新发展内资企业3750户、增长94.8%。农村土地确权登记颁证稳步推进，户籍登记实现了城乡统一。深化能源资源类产品价格改革，建立了政府债务管理和风险预警机制。

（七）推进社会治理创新，全力维护和谐稳定。抓住根本性和基础性问题，大力实施“治理创新工程”。扎实开展安全生产“三化”建设和隐患排查治理，开展“六打六治”专项行动，有效防范了较大以上事故发生。农民工工资清欠工作效果明显，帮助9600余名农民工追讨工资1.13亿元。围绕“抓三强、攻三案、提三率”，加大领导干部接访、约访、下访工作力度，信访形势总体平稳。狠抓普法宣传、依法治理、矛盾纠纷化解和特殊人群管理，平安汉中建设取得新成效。认真开展土地“双清”工作，顺利通过国家土地例行督察和年度卫片执法检查验收。食品药品监管有力，查办食品药品违法案件1261件。常态化、立体化落实治安防控措施，破获刑事案件6202起，查处治安案件9532起，社会大局保持稳定。

（八）践行为民务实清廉，加强政府自身建设。深入开展党的群众路线教育实践活动，驰而不息深化作风建设。下功夫清理办公用房、治理违规用车、遏制公款消费，全市“三公”经费下降29%，市本级下降41%。持续改文风、转会风，文件和会议数量分别下降8.6%和20%。自觉接受人大依法监督、政协民主监督，全年邀请省市人大代表、政协委员92人次列席政府重要会议，广泛听取意见建议；人大代表建议、政协委员提案办结率100%、满意率99%。公民代表进政府和大学生假期进机关活动富有成效。办理“市长信箱”来信、人民网留言2332件，办理回复率100%。市县行政服务中心受理审批事项54.9万件、办结率99%。在全省目标责任考核社会调查中，我市群众满意度居全省第二位。

审计、统计、档案、民族、宗教、人防、地震、气象、外事、侨务、对台、供销、地方志和国防动员等各项工作都取得新成绩。

各位代表，2014年市政府承诺的“十件实事”全部完成：建成农村公路567公里、标准化学校205所、农村互助幸福院120个、城乡体育惠民设施458处、基层文化活动场所433个，解决43.8万农村人口饮水安全问题，实现脱贫13.8万人，新农合和城镇居民医保政府补助标准提高到人均350元，汉江沿岸7个重点镇垃圾、污水处理设施开工建设，中心城区便民利民“10件好事”如期完成。

各位代表，过去一年，是我们直面经济下行压力，千方百计挖潜能、强动力、保增长，实现经济稳中有增、较快发展的一年；是我们主动顺应转型升级大势，齐心协力调结构、转方式、促长远，发展质量和效益同步提升的一年；是我们扎实开展群众路线教育实践活动，持之以恒转作风、促改革、惠民生，发展动力更加强劲、群众得到实惠更多的一年。成绩的取得，得益于省委、省政府和市委的坚强领导，得益于市人大、市政协的监督支持，得益于全市人民的顽强拼搏。在此，我代表市人民政府，向全市人民、驻汉部队指战员、武警官兵和公安干警致以崇高的敬意！向鼎力支持政府工作的各位代表、各位委员和社会各界人士表示衷心的感谢！

回顾过去一年工作，我们也清醒地看到汉中经济社会发展中存在的矛盾和问题：贫困人口多、发展底子薄、经济规模小仍是我们的基本市情，自主创新能力不强、产业结构不够优化、增长方式不尽合理仍是我们的发展短板，城镇化水平低、社会管理水平低、城乡居民收入水平低仍是我们的主要矛盾，交通制约、资金短缺、债务较大、环境限制仍是我们的发展瓶颈，少数政府工作人员进取意识不强、工作作风不实、服务效能不高等弊端，与群众的期待还有较大差距。这些问题，我们一定高度重视，认真加以解决。

二、新常态下我市发展的基本遵循和目标要求

2015年是全面完成“十二五”规划的收官之年，是全面深化改革的关键之年，也是全面推进依法治国的开局之年。去年底的中央经济工作会上，习近平总书记对我国经济社会发展形势作出了新常态的重要历史判断。做好今年的工作，要求我们必须在新常态的大逻辑下正视新挑战，把握新机遇，实现新发展。从新常态的要求看，汉中发展面临增速换挡、动力切换、结构优化和提质增效的多重任务，必须根据新的发展形势，转变工作思路，坚持调整存量与做优增量并举，充分利用“四化”互动的巨大潜力和发展空间，实现有质量有效益的中高速增长，促进经济社会发展行稳致远。从面临的挑战看，受宏观经济环境和各种矛盾的影响，经济下行压力仍然较大；调结构阵痛显现，转方式任重道远；中、省关于汉中功能区定位的刚性约束更趋严格，土地、环境、资金等经济发展的要素制约更为凸显；随着改革的深化，一些原有的社会矛盾和新的不稳定因素交错叠加，使稳定的任务更趋艰巨。我们必须保持定力、从容应对，综合施策、精准发力。从面临的机遇看，全面深化改革和依法治国的深入推进，将极大激发各类市场主体发展活力；国家加快“一带一路”和长江经济带建设，为汉中全方位开放、扩大招商引资拓展了新的空间；中央继续实施积极的财政政策和稳健的货币政策，为汉中加快发展提供了有利条件；随着汉中立体交通网络建设的推进，经济发展的资源优势和内在潜能将加速释放。总体看，新常态下机遇和挑战交织，希望与困难同在。必须切实把握汉中发展正处于追赶跨越、转型升级、潜能释放、民生普惠期的阶段性特征，准确认识新常态、积极适应新常态、主动引领新常态，推动新发展，再创新业绩。

2015年全市经济工作的总体要求是：深入学习贯彻中、省一系列重要会议和习近平总书记系列重要讲话精神，认真落实市委四届七次全会部署，以稳中求进为工作总基调，以“适应新常态、推动新发展”为主线，以提高经济发展质量和效益为中心，紧紧围绕“循环发展、生态宜居”的战略定位和“三市”奋斗目标，彰显“两汉三国、真美汉中”城市品牌，实施“六大战略”优化发展路径、壮大“五大产业”增强发展后劲、推进“十大工程”做实发展基础、建好“五大园区（村）”构筑发展平台，统筹做好稳增长、调结构、促改革、惠民生、防风险各项工作。

主要预期目标是：生产总值增长11.5%左右，固定资产投资增长23%以上，规模以上工业增加值增长17%左右，社会消费品零售总额增长13%左右，地方财政收入同口径增长12%以上，城镇居民人均可支配收入、农民人均纯收入分别增长11%、12%以上，城镇登记失业率控制在3.9%以内，居民消费价格指数控制在3%左右，单位生产总值能耗下降2.4%以上，化学需氧量、氨氮、二氧化硫、氮氧化物分别削减0.7%、1%、0.3%和7%以上。做好2015年工作，必须遵循以下原则：——牢牢把握中高速这一增长要求。从汉中欠发达这一基本市情出发，着眼如期全面建成小康社会宏伟目标，始终把发展作为第一要务，增强“不唯 GDP” 不等于“不要 GDP” 认识，保持高出全省平均水平1.5—2个百分点的增长速度。

——牢牢把握中高端这一调转取向。始终以提高经济发展质量和效益为中心，以推动经济结构战略性调整为抓手，改造提升传统产业，加快发展新兴产业，努力做到调速不减势、量增质更优，打造汉中经济升级版。

——牢牢把握促改革这一强劲动力。把改革创新作为主动适应和引领经济发展新常态的引擎，紧扣体制创新、科技创新两个关键，有的放矢、精准发力，释放改革红利，激活发展动力。

——牢牢把握保生态这一立市之本。强化生态立市、循环发展理念，加快构建走向生态文明新时代的空间格局、产业结构和生产生活方式，努力实现生态美、经济好、百姓富的有机统一。

——牢牢把握特色化这一发展路径。按照既定的县域发展定位，依托优势，因地制宜，分类指导，促进县区经济彰显特色、竞相赶超，错位发展。

——牢牢把握惠民生这一根本目的。更加注重保障基本民生，更加关注低收入群众生活，更加重视社会大局稳定，加快构建补缺兜底的社会保障体系，不遗余力地解民忧、谋福祉、促和谐。

三、2015年的重点工作任务

（一）狠抓项目建设，充分发挥投资拉动作用。坚持以重点项目为抓手，持续推进“千亿投资工程”，确保全社会固定资产投资突破1000亿元。

强力推进重点项目。坚持市级领导包抓、现场观摩点评、市县协调联动，确保汉中航空智慧新城建设取得突破性进展，镇巴区块油气资源勘探尽快下钻，西成客专、宝汉高速、阳安铁路扩能改造等项目快速推进，800千伏高压输电过境工程等项目开工建设，汉巴渝快速铁路、330千伏第二电源通道等项目前期工作步伐加快。全年实施重点项目335个，完成投资370亿元。

积极扩大招商引资。坚持向东招商与向西开放并举，加大点对点招商、网络招商、产业链招商、以商招商力度，着力引进投资规模大、带动效应好的重大项目和重点企业。下功夫解决投资落地、客商创业中存在的困难和问题，营造亲商、安商的良好环境。全年招商引资到位资金420亿元。

努力拓宽融资渠道。创新财政投入方式，用好政府与市场两种资源和手段，使财政资金扶持更加公平高效。引导金融机构扩大信贷投放，加大重点领域信贷支持。鼓励企业开展直接融资，在上海、陕西股交中心打造“汉中板块”。大力开展债券、信托等新型融资。创新项目融资理念，采取注入优质资产、盘活存量建设用地、股权社会化等多种方式筹集资金。加强金融市场监管，有效防范金融风险。

精心编制“十三五”规划。用新常态的大逻辑把握发展方位，加强对全局性、战略性重大问题的前瞻性研究，紧盯国家宏观调控趋向，着眼未来发展需要，抓紧编制“十三五”规划。特别要按照“补短板、增后劲、促均衡、上水平”的要求，策划一批彰显优势、优化结构、环境友好、关乎未来的大项目、好项目，为“十三五”发展提供有力支撑。

（二）突出提质增效，全面提升工业竞争力。启动实施新一轮“工业翻番工程”，加大结构调整和转型升级力度，促进工业经济速度效益再上新台阶。

大力培育市场主体。坚持市、县分层推进、接力扶持的方法，实施大众创业行动和“小巨人”成长计划，推动小微企业壮大入规、规上企业升级转型、非公有制经济持续繁荣，形成各类市场主体梯级成长的生动局面。全年新发展市场主体1.2万户，新培育规模以上企业30户、产值过亿元企业10户；非公经济增加值占生产总值的比重保持在51%左右。加快产业转型升级。围绕培育“五大产业”，全年实施重点技改项目75个、完成投资55亿元以上，建成汉王药业中药科技产业园和煎茶岭镍矿开发等项目，新开工秦元10万吨板状刚玉产业化、科瑞思矿业石英板材生产线等项目，加快实施陕飞扩能及大运飞机配套、金汉汽车零部件生产线等项目；抓好众泰汉中汽车产业化基地、洋县3万吨海绵钛等重大项目前期工作，争取早日开工建设。

提升工业园区水平。用好国家级开发区优惠政策，加快完善基础设施，加大企业引进力度，提升汉中经济开发区建设管理水平。坚持循环引领，强化扶持措施，完善功能配套，高标准建好汉江工业园区、洋县现代材料园等工业集聚区，促进资源整合、循环利用、链条延伸。全年县域工业园区完成投资145亿元，新入园企业30户，园区营业收入达到833.5亿元、增长18.5%以上。力促工业稳定增长。全面落实支持工业促销稳产保增长的各项政策措施，在电力直供、产销对接等方面加大支持，引导企业降低成本、提升效益，力争规模以上工业总产值增长18%。（三）注重转变方式，加快推进农业现代化。按照稳粮增收、提质增效、创新驱动的总要求，把转方式、调结构贯穿于“农业倍增工程”各个环节，更加注重农业科技创新，更加注重增强农业可持续集约发展能力，更加注重内部优化和内外联动，加快农业现代化步伐。

壮大主导产业。坚持稳定种植面积和推广良种良法并重，保证全年粮、油产量分别稳定在100万吨和17万吨以上。突出农业综合效益和竞争力提升，大力发展林下种养、冷水养殖和水果、药菌种植，做精做优茶产品，加快建设“十大绿色农产品生产基地”，提高特色优势农产品对“农业倍增工程”的贡献份额。全年生猪出栏420万头，茶叶、蔬菜、水果产量分别达3.4万吨、215万吨、43.5万吨。

发展新型主体。适应现代农业发展新趋势，积极培育职业农民，大力发展家庭农场，鼓励致富能人领办专业合作社和龙头企业，支持有条件的龙头企业开设专卖店、发展电子商务，不断拓展市场空间。全年新发展各类专业合作社220个，培育家庭农场100个、职业农民1000人。

建好现代园区。运用市场手段，引导和规范土地经营权有序流转，扩大园区规模和范围；捆绑整合涉农项目，加大科技、资本、人才等要素投入；拓展园区功能，创新管理体制，着力打造一批生产要素聚集、产业体系健全、科技支撑有力、经营方式先进、带动就业创业、综合效益显著的现代农业园区。

做优农业品牌。以品牌和标准化为引领，扩大有机、绿色、无公害农产品生产面积，加快国家地理标志产品认证步伐，打造一批驰名商标和知名品牌，新培育陕西名牌产品6个。要利用“一江清水供北京”这一纽带，加强衔接与推介，使更多的“汉”字牌高端农产品进入京津冀市场。

（四）做强全域旅游，带动三产快速增长。主动应对“高铁时代”带来的机遇和挑战，以“美丽中国·丝绸之路旅游年”活动为契机，强力推进“全域旅游工程”，带动第三产业发展提速、效益提高、水平提升。

做强旅游龙头。编制出台《全域旅游规划》，全年实施旅游重点项目18个，完成投资11.27亿元。加快龙岗文化生态旅游园、“一江两岸”文化园、留坝山地旅游度假示范区等项目建设，启动张骞文化旅游园区建设和华阳、紫柏山景区创5A工作，加快五龙洞、午子山、石门等景区创4A步伐。建成骆家坝红色旅游公路，开工建设华阳、黎坪旅游专线，规划建设一批城乡景区绿道慢行系统。加快竹园五星级酒店等星级饭店建设，抓好商务快捷酒店和乡村宾馆、农家乐更新改造，强化旅游市场管理，提高综合接待能力和服务水平。加强宣传促销，精心办好2015中国最美油菜花海旅游文化节等活动。全年旅游收入突破150亿元，增长15%以上。完善市场体系。加大高端化、智能化商业综合体引进力度，抓好亿丰国际商贸城、普汇中金世界港等项目，加快特色商业街区、商贸功能区和蔬菜流通、农产品冷链配送网络建设。全年实施商贸流通服务业项目43个、完成投资20亿元。引导做好“个转企”和大型专业批发市场集中收银工作，全年培育限额以上贸易单位30户。

繁荣消费市场。积极推广品牌连锁、物流配送、特许经营等现代流通方式，大力拓展电子信息、养老养生、大众餐饮、文化娱乐等消费业态，加快发展电子商务、会展经济和定制类消费，有效激发消费潜力。全年社会消费品零售总额完成318亿元。坚持综合施策，打好保障性住房、移民搬迁房、商品房互联互通的政策“组合拳”，促进房地产业健康发展。

发展外向型经济。主动融入“一带一路”建设，大力实施外贸龙头企业培育工程和外贸孵化器工程，组织企业高质量参加贸易投资洽谈活动，不断扩大外经外贸的贡献份额。全年外贸进出口总额增长10%以上。（五）统筹城乡发展，加快新型城镇化步伐。按照完善设施、提升管理、传承文化的基本要求，紧扣“建城、兴业、社保、管理”重点，持续推进“城乡统筹工程”。

高水平编制规划。根据新型城镇化的发展趋势、空间分布和“五大城市形象设计”，以城乡统筹、生态宜居为目标，科学确定新型城镇化的指标体系和发展路径，高水平规划城市公共服务基础设施体系。严格城市界线、生态底线、土地红线，促进城市规划与土地利用、产业发展、道路交通、管网管线、生态保护规划紧密衔接，加快形成总规管总、详规管块、专规管线、设计管点的城市规划体系，促进规划引领、约束作用的发挥。

分层次推进建设。中心城区建设上，实施36个城建重点项目、完成投资29亿元，积极推进滨江新区、兴元新区、江南片区等开发，加快东关正街、南关正街等历史风貌街区建设步伐，建好西新街东段、南一环西段等项目，建设一批与群众生活密切相关的市政设施，提升宜居指数和辐射带动能力。县城建设上，完善道路、供水供气、防洪排涝及污水、垃圾处理等配套设施，增强保障能力，提升发展品位。集镇建设上，重点推进铺镇、青木川、骆家坝等重点镇、文化旅游名镇建设，努力打造一批宜业宜居的大镇名镇。村庄建设上，按照“规划先行、分类指导、产村融合、绿色家园”的要求，大力推进美丽乡村、新型农村社区建设。

均等化提升服务。消除影响平等就业的制度障碍，大力拓展城镇就业空间。加快推进教育、医疗、养老、社保等制度改革，提高基本公共服务均等化水平。从规范管理行为和社会活动细节入手，解决好交通拥堵、市容不整、公共服务盲点多等问题。巩固“国卫”创建成果，持续开展省级“环保模范城市”、“生态文明城市”和国家级“园林城市”创建工作，不断提升以人为核心的新型城镇化水平。

（六）严格环境保护，持续推进生态文明建设。大力推进汉江流域污染防治三年行动计划，坚定不移地实施“生态环境工程”，保护好汉中的青山蓝天净水。

加快发展循环经济。认真落实市委、市政府关于加快推进循环经济发展的实施意见，严格项目环评准入制度，大力推进资源循环高效利用和无害化生产，促进产品之间、企业之间、产业之间和社会各领域之间的大小循环，做到能源梯级利用、水资源循环利用、废物交换利用、土地集约利用，提高资源能源利用水平。全年实施循环发展项目78个，完成节能项目7个。

强力整治环境污染。下功夫治理钢铁、水泥、冶炼、建筑施工等行业污染，启动和推进中材水泥、南化、啤酒厂搬迁和各县小化工、小建材淘汰、转迁工作。抓好“煤改气”、“油改气”和“黄标车”淘汰工作，实施一批电热锅炉、电源热泵和电动汽车等清洁能源项目，继续做好秸秆禁烧和综合利用，解决好燃煤锅炉烟尘、餐饮油烟排放、烟花爆竹燃放、道路及施工扬尘、机动车尾气排放等污染问题，力争全年空气质量优良天数达到省上考核要求。加大医药化工、食品酿造和尾矿库等行业废水治理，抓好危险废物、重金属和农村面源污染防治，继续开展垃圾集中清运活动，推进县城污水管网完善配套和正常运行，加快沿江重点镇“两厂（场）”建设，确保汉江出境水质保持在二类以上标准。

推进生态工程建设。继续抓好天保工程，完成省定退耕还林任务；开展重点流域生态治理，加快南郑县、洋县、勉县、西乡县农村环境连片整治示范项目建设，力争全市纳入中省连片整治示范市建设计划；大力拓展城镇绿化空间，均衡绿地分布，扩大绿地总量。全年造林20万亩，实施街道、交通干线及河堤绿化380公里，治理水土流失850平方公里；谋划启动一批江河湿地建设项目。

（七）做实社会事业，全力保障和改善民生。统筹推进“民生改善工程”、“文化振兴工程”和“扶贫攻坚工程”，努力做好兜底线、补短板、保基本各项工作。

完善社会保障体系。开展全民创业活动，多措并举促进高校毕业生、退役军人、城镇困难人员、失地农民创业就业，全年新增就业3.2万人，转移农村富余劳动力76万人。动态监测城乡居民收入变化，适时出台促进居民增收的指导性政策。继续做好个体工商户、灵活就业人员参加社会保险工作。围绕托底线、救急难、可持续，抓好社会救助体系建设，加大临时救助、医疗救助力度。全面落实孤儿、残疾人、老年人优待政策。

推进教育科技卫生工作。深化教育体制改革，优化学校布局，持续推进“双高双普”；抓好第二轮学前教育“三年行动”和中心城区学校建设“三年攻坚计划”实施，公办幼儿园要占到幼儿园总数的38%，义务教育阶段教师、校长轮岗率不低于5%；组建市级职业教育集团，规范民办教育，继续抓好移民搬迁家庭适龄学生免费接受高等职业教育工作。加快科技资源整合，支持企业成为研发主体，建设一批技术研发中心、创新服务平台、科技示范园区，提升科技对经济的贡献份额。深化公立医院改革，优化医疗资源结构和布局，加快组建医疗联合体，建立三级医院对口帮扶和村医培训、退出机制；放宽社会力量办医准入，支持民营医疗机构发展。持续稳定适度低生育水平，人口自然增长率控制在5‰以内。繁荣文化体育事业。积极培育和弘扬社会主义核心价值观，传播主流文化，培育文明风尚。加强公民道德建设，引导公民自觉履行法定义务、社会责任、家庭责任。把法治教育纳入精神文明创建内容，开展群众性法治文化活动，提高全民法治意识。大力实施文体惠民工程，深入开展全民健身和公共文化活动，加快市体校、羌文化中心、青少年宫等公共文体设施建设。精心打造“汉中栈道”、“汉使张骞”、“古路坝灯火”等文化精品，尽快组建汉中文化传媒集团，抓好“两汉三国、真美汉中”文化景区项目建设。文化产业增加值增长10%以上。抓好民生重点工程。加大连片开发、产业扶贫、精准扶贫力度，全年实施整村推进连片开发项目村146个。以国省干线改造、市内外区域互联互通项目和镇村公路升等改造为重点，开展“交通重点建设年”活动。坚持规范有序、一户一宅推进，加快农村土坯房改造。统筹解决好资金、用地和政策创新难题，全年新开工保障性住房2万套，其中棚户区改造1.6万户；完成陕南移民搬迁2.2万户；新修、加固汉江干支流堤防30公里。

提升社会治理水平。持续推进“治理创新工程”，常态化抓好安全生产隐患排查治理和打非治违，全力遏制较大事故，坚决杜绝重特大事故；加强基层防灾减灾体系建设，提升应急处突能力；强化全过程监管，保障群众食品和用药安全；依法规范信访行为，完善人民调解、行政调解、司法调解联动工作体系，加强信访维稳网络建设，抓好领导干部“四访”和积案化解工作，有效化解各类矛盾纠纷；持续推进平安汉中建设，切实维护社会稳定。

（八）加快改革步伐，释放红利激发活力。把全面深化改革作为推动经济社会发展的关键措施，以重点领域和关键环节的改革突破带动全面改革，加快释放制度红利。

深化经济体制改革。围绕激发市场主体活力，完善非公经济发展机制，有序放开市场准入，引导民营企业依法进入垄断领域，进入社会需要而产品与服务短缺的领域；大力发展混合所有制经济，鼓励社会资本参与国有资本重组和投资，激活国有资产存量；深化财政管理绩效综合评价改革，推进“营改增”、消费税、资源税等税制改革；将政府性债务纳入预算管理，加大财源建设力度、培育新的增长点；建立镇村土地流转服务平台，稳步推进农村集体建设用地“同权同价”入市交易，加快构建立体式、复合型的现代农业经营体系。

深化行政体制改革。抓住简政放权这一关键，重点抓好市县机构改革，稳妥推进镇村综合改革和村委会换届，加快事业单位分类改革；深化行政审批制度改革，全面清理非行政许可审批事项，对保留的审批、收费事项一律实行目录清单管理；深化商事制度改革，进一步放宽市场主体登记条件，实行“注册资本金认缴”、先照后证，允许“一照多址”或“一址多照”；健全社会信用、信息共享、企业年报公示和经营异常名录等制度体系，依法规范市场主体经营行为；稳步推进公车改革。

深化社会领域改革。着眼提升社会治理能力，启动机关事业单位养老保险制度改革，加快构建统筹城乡的养老保障体系；深化医疗卫生体制和药品流通体制改革，着力解决小医院难看病、大医院看病难和药品配送不到位问题；加快构建以企业为主体的创新体系，推动科技、资源、金融相互融合；完善实体市场和网络交易全领域、全过程监管体系，织牢织密监管网底；巩固提升食品药品监管体制改革成果，加快检验检测和诚信体系建设；推进社会组织登记改革，发挥好社会组织的积极作用。

各位代表，今年市政府继续为全市人民办好十件实事：一是将企业退休人员基本养老金标准提高10%、居民基础养老金人均最低标准提高15元；二是将城市低保标准每人每月提高35元，将农村低保、农村五保集中供养、分散供养标准分别提高到每人每年2250元、6000元、5500元；三是新建农村互助幸福院等各类养老服务机构120个，扶持发展民办养老机构10个、城市日间照料中心及养老服务站20个；四是建设标准化学校138所；五是实现脱贫13万人；六是解决18万人饮水安全问题；七是完成居民用电“一户一表”改造3万户；八是实现100%的镇通油路或水泥路；九是改造农村土坯房3000户；十是办好中心城区便民利民10件好事。

四、努力建设人民满意的法治政府

发展的重任，人民的期待，对政府工作提出了新的更高的要求。我们将认真贯彻党的十八届四中全会精神，围绕职能科学、权责法定、执法严明、公开公正、廉洁高效、守法诚信的目标，加快建设人民满意的法治政府。

全面履职，增强落实力。坚持用法治思维和法治方式履行职责，加快职能转变，推行政府权力清单、责任清单、负面清单、收费清单制度，切实做到法定职责必须为、法无授权不可为。创新审批方式，简化前置条件，推行并联审批，提高服务效能。弘扬担当精神，遇到难题敢闯敢试，直面矛盾敢于碰硬，面对风险敢于担责。严格责任管理，坚持首问负责、按期办结、督查考核和责任倒查追究，坚决纠正不为、乱为，坚决克服懒政、怠政，坚决惩处失职、渎职，进一步提高政府效能。

规范运行，彰显公信力。落实公众参与、专家论证、风险评估、合法性审查、集体讨论决定的决策机制，确保政府决策制度科学、程序正当、过程公开、责任明确。坚持以公开为常态、不公开为例外，重点推进财政预算、公共资源配置、重大项目审批和实施、社会公益事业建设等领域信息公开，加快实施行政执法公示制和政府规范性文件“三统一”；广泛开展公民代表进政府和大学生假期到政府见习活动。自觉接受市人大法律监督、工作监督和政协民主监督，认真办理人大代表建议和政协委员提案，重视司法监督、舆论监督和社会公众监督，对公共资金、国有资产管理等实行审计全覆盖，让权力在阳光下运行。

严格执法，提升执行力。全面落实行政执法责任制，完善执法程序，加强执法监督，构建指挥令行禁止、运转高效灵活、执行简便有力的综合行政执法模式。建立健全行政裁量权基准、重大执法决定法制审核、行政执法人员资格管理和持证上岗、执法全过程记录等制度，切实提高执法和服务水平。加大食品药品、环境保护、安全生产等重点领域执法力度，切实维护人民群众切身利益。

勤廉为民，强化凝聚力。巩固和拓展群众路线教育实践活动成果，严守党的纪律和规矩，严格执行八项规定，以“三严三实”为言行准则，勤勉履职、廉洁自律，学会在纪律严格约束下工作，在社会普遍监督下干事，在全面依法行政中作为。厉行勤俭节约，反对铺张浪费，严格控制一般性支出，确保“三公”经费只减不增。不折不扣落实党风廉政建设主体责任，认真履行“一岗双责”，促进政府工作人员筑牢思想防线、守住道德底线、不越纪律红线，以为民务实清廉的作风取信于民、凝聚合力。

各位代表，新常态赋予新使命，新起点开启新征程。让我们在市委的坚强领导下，紧紧依靠全市人民，埋头苦干，开拓进取，全面完成“十二五”经济社会发展任务，共同谱写汉中“三市”建设的新篇章！
